# Narciclasine inhibits phospholipase A2 and regulates phospholipid metabolism to ameliorate psoriasis-like dermatitis

**DOI:** 10.3389/fimmu.2022.1094375

**Published:** 2023-01-04

**Authors:** Yi Kong, Jian Jiang, Yuqiong Huang, Xin Liu, Zilin Jin, Li Li, Fen Wei, Xinxin Liu, Jie Yin, Yonghui Zhang, Qingyi Tong, Hongxiang Chen

**Affiliations:** ^1^ Department of Dermatology, Union Hospital, Tongji Medical College, Huazhong University of Science and Technology, Wuhan, Hubei, China; ^2^ Department of Dermatology, The Second Affiliated Hospital of Xi’an Jiaotong University, Xi’an, Shanxi, China; ^3^ Department of Dermatology, Union Shenzhen Hospital, Huazhong University of Science and Technology, Shenzhen, Guangdong, China; ^4^ Hubei Key Laboratory of Natural Medicinal Chemistry and Resource Evaluation, School of Pharmacy, Tongji-Rongcheng Center for Biomedicine, Tongji Medical College, Huazhong University of Science and Technology, Wuhan, Hubei, China

**Keywords:** psoriasis, narciclasine, lipid metabolism, phospholipase A2, keratinocyte

## Abstract

**Introduction:**

Psoriasis is a common inflammatory skin disease recognized by the World Health Organization as "an incurable chronic, noninfectious, painful, disfiguring and disabling disease." The fact that metabolic syndrome (MetS) is the most common and important comorbidities of psoriasis suggests an important role of lipid metabolism in the pathogenesis of psoriasis. Narciclasine (Ncs) is an alkaloid isolated from the Amaryllidaceae plants. Its biological activities include antitumor, antibacterial, antiinflammatory, anti-angiogenic and promoting energy expenditure to improve dietinduced obesity. Here, we report that Ncs may be a potential candidate for psoriasis, acting at both the organismal and cellular levels.

**Methods:**

The therapeutic effect of Ncs was assessed in IMQ-induced psoriasis-like mouse model. Then, through in vitro experiments, we explored the inhibitory effect of Ncs on HaCaT cell proliferation and Th17 cell polarization; Transcriptomics and lipidomics were used to analyze the major targets of Ncs; Single-cell sequencing data was used to identify the target cells of Ncs action.

**Results:**

Ncs can inhibit keratinocyte proliferation and reduce the recruitment of immune cells in the skin by inhibiting psoriasis-associated inflammatory mediators. In addition, it showed a direct repression effect on Th17 cell polarization. Transcriptomic and lipidomic data further revealed that Ncs extensively regulated lipid metabolismrelated genes, especially the Phospholipase A2 (PLA2) family, and increased antiinflammatory lipid molecules. Combined with single-cell data analysis, we confirmed that keratinocytes are the main cells in which Ncs functions.

**Discussion:**

Taken together, our findings indicate that Ncs alleviates psoriasiform skin inflammation in mice, which is associated with inhibition of PLA2 in keratinocytes and improved phospholipid metabolism. Ncs has the potential for further development as a novel anti-psoriasis drug.

## Introduction

Psoriasis is a common chronic inflammatory skin disease that affects 2%-11% of the global population (1). Its pathogenesis is complex and has not been fully elucidated. In response to infection, trauma, ultraviolet light and genetic susceptibility to psoriasis, keratinocytes are activated with increased release of proinflammatory cytokines. In addition, chronic activation of the Th17-centered adaptive immune system can lead to long-term damage not only to the skin but also to other tissues and organs (2). Metabolic syndrome (MetS) is one of the most common and important comorbidities of psoriasis (3, 4), suggesting an important role of lipid metabolism in the pathogenesis of psoriasis. Phospholipase A2 (PLA2) enzymes catalyze the hydrolysis of glycerophospholipids to produce proinflammatory lipid mediators and free fatty acids. These bioactive lipids are critical in maintaining the epidermal barrier and immune homeostasis during inflammation and disease (5). PLA2 enzymes are classified into multiple families, and perturbation of specific PLA2-driven lipid pathways has been implicated in inflammation and allergic skin diseases (6). The most studied and best understood in skin homeostasis is PLA2G2F. It has been shown to be overexpressed in hyperproliferative epithelial diseases (7). By driving a unique lipid pathway, PLA2G2F promotes the development of psoriasis, contact dermatitis and skin cancer (7). Furthermore, inhibition of PLA2G4D/E was found to limit abnormal keratinocyte differentiation and inflammatory responses (8). These studies provide clear evidence to support the potential of controlling inflammatory lipid mediators through PLA2 inhibition as a treatment for psoriasis.

Natural products have been an important source of small molecule drugs for clinical use. Narciclasine (Ncs) is an isoquinoline alkaloid found in the Amaryllidaceae family of flowering plants. Since Narciclasine was isolated, it has been widely studied as a promising antitumor drug because of inhibitory effect on cell proliferation (9–14). Subsequently, its anti-inflammatory effect was gradually discovered. In a rat arthritis model, Ncs was found to effectively prevent paw swelling and reduce ex vivo proinflammatory cytokines production in PMBC, splenocytes, and DLN cells (15–17). In addition, recent studies have shown that Ncs can regulate energy metabolism, attenuate diet-induced obesity (DIO) in mice by promoting energy expenditure and improve insulin sensitivity (18). Despite the potent anti-tumor, anti-inflammatory and improving metabolism properties of Ncs, no studies have investigated its possible role in psoriasis. In this study, we determined the therapeutic effect of Ncs on imiquimod (IMQ)-induced psoriasis-like skin inflammation and explored the possible mechanisms. We found that Ncs significantly improved psoriatic skin lesions, inhibited keratinocyte proliferation and exhibited a direct inhibitory effect on Th17 cell polarization. Integration of Transcriptomic and lipidomic data attributed the beneficial effect of Ncs on psoriasis to the extensive regulation of phospholipid metabolism-related genes, especially the PLA2 family, and the amelioration of lipid metabolic disorders in IMQ-induced psoriatic mice.

## Methods

### IMQ induced psoriasis-like mouse model

All animal experiments were conducted according to the guidance of China Animal Welfare Legislation, and approved by the ethical institutional review board of Union hospital, Huazhong University of Science and Technology. 6-8 weeks old mice (female BALB/C background) purchased from Hunan SJA Laboratory Animal Co., Ltd., were housed in a constant temperature and humidity with a 12:12 h dark/light cycle. Mice were randomly divided into 3 groups (n = 5 per group) and shaved on the back skin. Apply imiquimod cream (Sichuan Mingxin Pharmaceutical Co., Ltd., No. 120503, Sichuan, China) 62.5mg topically on the shaved area for 6 consecutive days to establish a psoriasis-like lesion model. Narciclasine was purchased from Medchemexpress (MCE, HY-16563) and was prepared from a solution containing 2% DMSO and 5% Castor Oil at a dose of 2mg/kg/day. In the IMQ and Ncs group: 2 days before IMQ administration, mice were intradermal injected with solvent control or Ncs solution on the back until the end of the study. Con group: daily intradermal injection of solvent on the back as control and topical vaseline treatment. Intradermal injection was performed in the shaved area on the back of the mice. Avoid injecting at the same site every day to minimize skin damage caused by the operation. All injection procedures should be performed at least 4 hours apart from drug administration. The body weight and PASI score of the mice were recorded every day of the experiment. On day 7, the mice were sacrificed and their blood, skin, and spleen were collected for further analysis.

### HE and immunohistochemical analysis

The skin tissue was fixed with 4% paraformaldehyde and embedded in paraffin. Paraffin sections were stained with hematoxylin and eosin (HE) and examined under a microscope. For Ki67 and CD4 staining, antigen repair was first performed with sodium citrate/EDTA antigen repair solution (Servicebio Biotechnology, Wuhan, China). After blocking, the sections were incubated overnight at 4°C with anti-Ki67 (Servicebio Biotechnology, GB111141) and anti-CD4 antibody (Abcam, ab183685). The next day, the sections were treated with the corresponding secondary antibodies for one hour, and the immunostaining results were observed under a microscope (Olympus, Japan). The integrated optical density (IOD) of Ki67+ (in epidermal region) or CD4+ cells in the skin was calculated using ImageJ software.

### Quantitative real-time PCR tests

Total RNA was extracted from murine skin tissues or cells with TRIzol reagents (Thermo Fisher, USA, Cat: 15596026) and was transcribed to cDNA using HiScript QRT SuperMix reverse transcriptase (Vazyme, R223–01, Nanjing, China). Quantitative PCR was then performed on ABI QuantStudio 5 (Thermo Fisher Scientific, USA) using SYBR Green qPCR Mix (Vazyme Biotech Co.,Ltd, China). The assessment of αTubulin was used as an internal control and the 2-ΔΔCT method was utilized to quantitatively analyze the data. Primer sequences are shown in **Supplementary Table S1**.

### Cell culture

HaCaT cells (purchased from ATCC) were cultured in DMEM (Gibco) supplemented with 10% fetal bovine serum (FBS, Gibco) and 1% penicillin/streptomycin.

### Cell proliferation assays

Cell viability was detected by CCK8 assays. HaCaT cells were seeded in 96-well plates at a density of 3×10^3^ per well. After 24, 48, 72, or 96 hours of incubation, the cells were co-cultured with 10% CCK8 solution for 2 hours at 37° C. Then the absorbance at 450 nm was measured using a spectrophotometer microplate reader.

### Apoptosis and cell-cycle analysis

HaCaT cells were cultured for 48h with or without 0.1um Ncs in the presence of 10ng/ml LPS. Cells were harvested and stained with FITC Annexin V and PI according to the manufacturer’s instructions (ABP Bioscience, Wuhan, China) and then analyzed by flow cytometry (Accuri C6, Becton Dickinson).

For cell-cycle analysis, cells were harvested and fixed in 100% ethanol and stained with propidium iodide. Events were collected by flow cytometry (Accuri C6, Becton Dickinson) and cell-cycle distribution was analyzed using ModFit software.

### Western blotting

The total protein from the skin of each mouse was lysed in RIPA lysis buffer (Beyotime, P0013D, Shanghai, China) containing PMSF (Beyotime, ST506, Shanghai, China) and phosphorylated protease inhibitors (Beyotime, P1081, Shanghai, China), and 30 ug of total protein was used for each blot. The samples were separated by SDS-polyacrylamide gels and then transferred onto a nitrocellulose filter membrane (NC, Millipore, USA). After closure of the blocking solution for 1 h, the membranes were incubated overnight with 1:1000 dilutions of anti-STAT3(CST, #9139, Boston, American), p-STAT3 (CST, #9145, Boston, American), CDK1 (Proteintech, Cat No. 19532-1-AP, China), CDK2 (Proteintech, Cat No. 10122-1-AP, China), CyclinA2 (Proteintech, Cat No. 18202-1-AP, China), CyclinB1 (Proteintech, Cat No. 55004-1-AP, China) and αTubulin (Proteintech, Cat No. 11224-1-AP, China) primary antibodies. After washing with TBST, the membranes were incubated with the secondary antibodies anti-rabbit IgG (H+L) (DyLight™ 800, Cell Signaling Technology, USA) at a 1:30,000 dilution and then imaged using a LiCor Odyssey scanner (LI-COR, USA).

### Flow cytometry

Mouse spleens were collected, ground and filtered through a sieve to obtain single cell suspensions. To detect Th1, Treg and Th17, cells were stimulated with phorbol myristate acetate (PMA)/ionomycin mixture and GolgiPlug (BD Biosciences) for 4h. Anti-CD4-FITC (BD Biosciences, 553046), Anti-CD3e APC-Cy7 (BD Biosciences, 557596) and Anti-CD25-BV421 (BD Biosciences, 607180) were used to stain the surface markers. After washing, cells were fixed and permeated using the Fixation/Permeabilization Kits (eBioscience). Anti-Foxp3-Alexa 647 (BD Biosciences, 560401), Anti-IFNG-PE-Cy7 (BD Biosciences, 557649), Anti-IL17A-PE (BD Biosciences, 559502) antibody was used for staining of intracellular markers. Finally, stained cells were detected by flow cytometry (FACSAriaIII, BD Biosciences) and data were analyzed with Flowjo software.

### *In vitro* T cell differentiation

Naive CD4^+^ T cells were purified from spleen single cell suspensions using Mouse Naive CD4^+^ T Cell Isolation Kit II (Miltenyi Biotec). 24-well plates were coated with anti-CD3 (5 ug/ml, Leinco) at 37°C for 1 h. After washing, purified naive CD4^+^ T cells were cultured with anti-CD28 antibody (2 ug/ml, Leinco) for 5 days. For Th17 polarization, TGF-β (2 ng/ml, PeproTech), IL-6 (30 ng/ml, PeproTech), IL-1β (10 ng/ml, PeproTech), and IL-23 (20 ng/ml, R&D Systems) were used for cell activation in the presence of anti–IFN-γ (10 ug/ml, Leinco) and anti–IL-4 (10 ug/ml, Leinco).

### RNA-sequencing

Three biological replicates for the Con, IMQ and Ncs group were sequenced by Frasergen Bioinformatics Co., Ltd. (Wuhan, China) using the MGI-SEQ 2000 platform. Normalization and differential expression analysis were performed by DESeq2 with the criteria in the program: Fold change > 2-fold, p value < 0.05, False Discovery Rate (FDR) < 0.05. The visualization of KEGG pathway enrichment analysis was realized by OmicShare cloud platform (https://www.omicshare.com/).

### Lipidseq

25 mg of mouse skin tissue was weighed and ground in 800 ul extraction buffer (dichloromethane/methanol = 3:1, v/v, pre-cooled at -20°C) for 5 min. After sonication in ice bath for 10min, samples were placed in the refrigerator at -20°C overnight. After centrifugation at 25,000g, 4° C for 15min, 600µL of the supernatant was taken out for lyophilization, and then redissolved in solvent (isopropanol/acetonitrile/water = 2:1:1). To assess the repeatability and stability of the LC-MS analysis, 20 uL of each sample was mixed into a quality control (QC) sample.

The LC–MS analysis was carried out with Waters UPLC I-Class Plus (Waters, USA) equipped Q Exactive High Resolution Mass Spectrometer (Thermo Fisher Scientific, USA). A CSH C18 column (1.7 um, 2.1 × 100 mm, Waters, USA) was selected for UPLC analysis. A linear gradient consisting of 0.1% formic acid in water (mobile phase A) and acetonitrile (mobile phase B) was used with gradient program. The injection volume was 5 uL, the flow rate was 0.4 mL/min, and the column temperature was maintained at 55°C. Primary and secondary mass spectrometry data was collected using a Q Exactive mass spectrometer (Thermo Fisher Scientific, USA). The electro spray source (ESI) detection was operated in the multiple reaction monitoring (MRM) mode. Detailed ion source (ESI) parameter settings include: 40 L/min of sheath gas, 10 L/min of aux gas, 3.80 (|KV|) spray voltage in the positive ion mode and 3.20 (|KV|) in the negative ion mode, capillary temperature (320°C), and aux gas heater temperature (350°C). The mass spectrometry data were collected by LipidSearch v.4.1 (Thermo Fisher Scientific, USA) software. After analysis, a data matrix containing lipid molecule identification and quantification was obtained.

The original data were further analyzed and filtered using metaX software. The data were processed by supervised partial least squares discriminant analysis to obtain group clustering. Fold change (FC) was detected using univariate analysis and t test analysis was performed to calculate P value. Differential metabolites between the two groups were based on |FC| >=2 and P<0.05.

### ScRNA-Seq data analysis

The original scRNA-seq dataset was obtained from the GEO database, consisting of skin samples from three psoriasis patients and three healthy volunteers (GSE162183). scRNA-seq data analysis was performed using the Seurat R package (version 4.0.5). Data were filtered for UMIs less than 200 and greater than 25% mitochondrial expression. Variable genes were detected using the Seurat function FindVariableGenes. PCA was used to reduce the dimensionality of the cells, and the FindClusters function was used to cluster the cells with a resolution of 0.1. Visualized clusters are generated by UMAP. The cell types of each cluster were defined according to the signature genes in the original text (19). Differential genes in each cell type or group were visualized using DotPlot, FeaturePlot, and VlnPlot.

### Statistical analysis

Statistical analysis was performed by GraphPad Prism 8.0 software. Data were presented as the mean ± SEM. Statistical comparisons were determined using Student’s t-test and differences were considered statistically significant at P < 0.05.

## Results

### Ncs alleviates IMQ-induced psoriatic dermatitis

**Figure 1:**
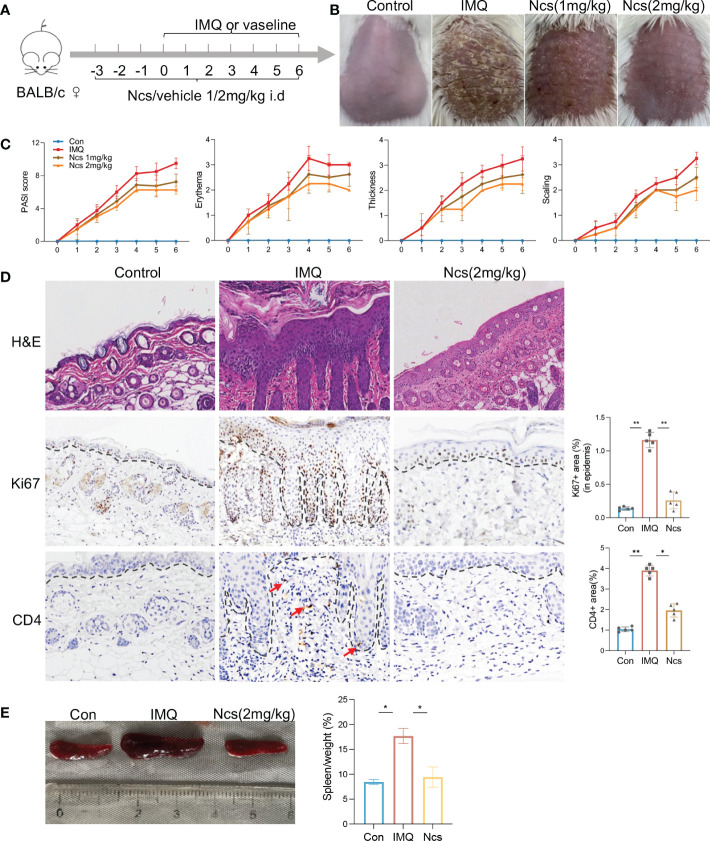
Ncs alleviated IMQ-induced psoriatic skin inflammation in mice. **(A)** Mice were pretreated with Ncs or vehicle for 3 days by intradermal injection. Imiquimod or vaseline cream was introduced in day 0 and mice were sacrificed in day 6. **(B)** Phenotypic presentation of lesional skin in day 6 (one representative mouse given, n = 5). **(C)** Clinical scores for erythema, scaling, and thickness of the dorsal skin were counted daily, and the PASI score was calculated by summing the scores from the three independent criteria. **(D)** H&E staining and immunohistochemical determination of Ki67+ and CD4+ cell infiltration in skin tissue of each group. Magnification, 200X. For counting Ki67+ (in epidermis) and CD4+ area, five fields in each section of each sample were calculated. **(E)** Representative pictures of the spleen from the indicated group and spleen index (spleen weight as a percentage of body weight) was calculated on day 6. Data are representative of three independent experiments with 5 samples per group in each. Data are presented as the mean ± SEM. P-values were determined using t test by Prism 8; *p < 0.05; **p < 0.01.

To evaluate the potential therapeutic role of Ncs in psoriatic skin inflammation, we used a mouse model of IMQ-induced psoriasis treated with Ncs. As shown in **Figure 1A**, BABL/C mice were randomly divided into 3 groups: Con group was given daily intradermal injection of placebo, IMQ group and Ncs goup was given daily intradermal injection of placebo/Ncs (1mg/kg and 2mg/kg) as well as topical application of IMQ. During the experimental period, the IMQ group developed typical scales, thickness, and erythema on the back skin. Application of Ncs significantly reversed the severity of lesions and was accompanied by a reduced PASI score. (**Figures 1B, C**). The dose of 2mg/kg was used for the subsequent experiments since it demonstrated greater improvement in psoriasiform dermatitis than that of 1mg/kg. Histopathological results showed that Ncs treatment improved epidermal thickening and immune cell recruitment. (**Figure 1D**). Immunohistochemical staining for Ki-67 and CD4 also demonstrated decreased epidermal hyperplasia and CD4^+^ T cell infiltration in dermis (**Figure 1D**). As expected, the expression of various psoriasis-related inflammatory molecules in the skin and serum of mice treated with Ncs was significantly decreased compared with the IMQ group (**Figure S1**). Notably, the spleen index (spleen weight to body weight ratio), which is an indicator of the severity of inflammation, was reduced in Ncs-treated mice (**Figure 1E**). Taken together, these results suggested the therapeutic effect of Ncs on IMQ-induced psoriatic skin lesions.

### Ncs has potent anti-inflammatory properties against psoriasis *in vitro* and *in vivo*

**Figure 2:**
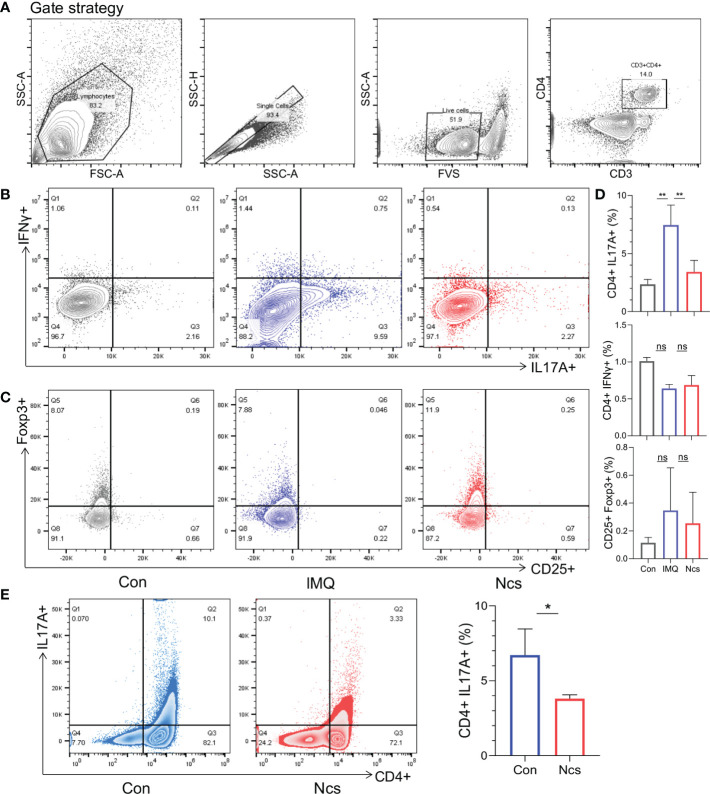
Ncs reduced the proportion of Th17 cells in the spleen of IMQ-induced psoriasis-like mice and inhibited Th17 polarization *in vitro*. As described above, intradermal injection of Ncs(2mg/kg) and topical administration of IMQ were performed on the back of mice. After the mice were sacrificed on day 6, spleens were extracted and dissociated into single-cell suspension for flow cytometry. **(A)** The gating strategy of different T cell subtype. **(B, C)** Representative flow cytometric analysis of Th1 (CD4+IFNγ+), Th17 (CD4+IL17A+), and Treg (CD25+Foxp3+) cells in splenic CD4+ T cells from each group (n=3). **(D)** Statistical data are shown in the histogram. **(E)** Naïve CD4+ T cells from mouse spleen (n=3) were stimulated with anti-CD3 and soluble anti-CD28 under Th17-polarizing condition with or without Ncs *in vitro*. Five days later, the percentages of CD4+ and Th17 (CD4+IL17A+) cells were detected by flow cytometry and statistical data are shown in the right panel. Flow data are representative of three independent experiments with 3 samples per group in each. Data are presented as the mean ± SEM. P-values were determined using t test by Prism 8; *p < 0.05; **p < 0.01; ns, no significance.

According to the results presented above, compared with the IMQ group, the spleen size of Ncs-treated mice returned to that of the control group, suggesting a preliminary effect of Ncs on immune system. To further investigate the changes in the proportion of spleen immune cells, flow cytometry was used to verify the frequency of Th subtypes in single cell suspensions isolated from the spleen among different groups. Compared with the control group, the frequency of CD4^+^IL17A^+^(Th17) cells was higher in the IMQ group and recovered after Ncs treatment (**Figures 2A, B**), while the percentage of CD4^+^IFNγ^+^(Th1) cells and CD4^+^ CD25^+^ Foxp3^+^(Treg) cells was not statistically different (**Figures 2B-D**). Moreover, Ncs lowered the percentage of total CD4+ T cell in the spleen (**Figure S2**). We next studied the effect of Ncs on Th17 cell polarization. Mouse Naive CD4^+^ T cells were polarized under Th17 condition for 5 days *in vitro* with or without Ncs. We evaluated the frequency of induced Th17 cells and found a significant decrease in the proportion of CD4^+^ IL17^+^ cells (**Figure 2E**). These data suggested that Ncs inhibits Th17 development and thus suppress immune activation in psoriasis.

**Figure 3:**
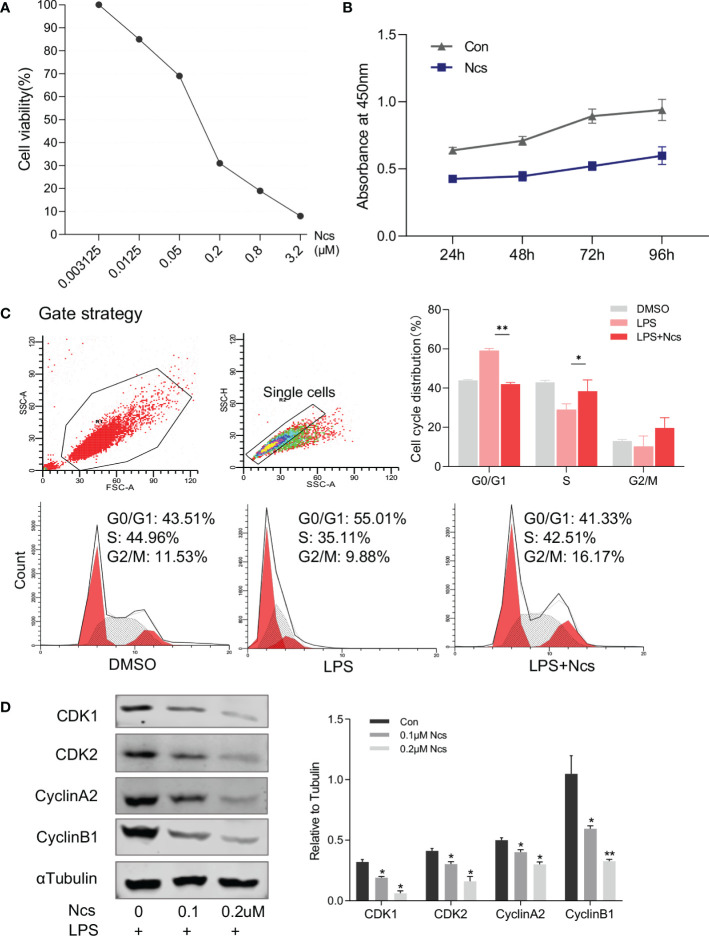
Effects of Ncs on proliferation and cell cycle of HaCaT cells. **(A)** HaCaT cells were cultured for 48h with gradient concentration of Ncs, and absorbance at 450nm was detected by CCK8 assay and cell survival rate was calculated. **(B)** LPS-primed HaCaT cells were treated with or without 0.1um Ncs for 24/48/72/96h, absorbance at 450nm was measured by CCK8 assay. **(C)** LPS-primed HaCaT cells were treated with or without 0.1um Ncs for 24h. Cell cycle distribution was analyzed by flow cytometry, with summary bar charts showing the percentage of cells in each phase. **(D)** Expression of cell cycle-related protein and quantification was evaluated by western blotting. Each experiment was repeated three times in HaCaT cells. Data are presented as the mean ± SEM. P-values were determined using t test by Prism 8; *p < 0.05; **p < 0.01.

Abnormal proliferation and apoptosis of keratinocytes is a major characteristic of psoriasis skin lesions. We next investigated the effect of Ncs on keratinocytes. CCK8 assay showed that the inhibition of HaCaT cell proliferation by Ncs was concentration- and time-dependent (**Figures 3A, B**). Since the complex formed by cyclin and cyclin-dependent kinase (CDK) plays an important role in cell cycle regulation, we examined the changes in HaCaT cell cycle to determine the mechanism of reduced proliferation by Ncs. It was found that Ncs treatment increased the proportion of HaCaT cells in S phase of the cell cycle (**Figure 3C**). Western blot also showed that Ncs reduced the expression of CDK1, CDK2, cyclinA2, CyclinB2 (**Figure 3D**), suggesting that Ncs inhibits keratinocyte proliferation by inducing cell cycle arrest. In addition, Ncs treatment slightly promoted HaCaT cell apoptosis, although not statistically significant (**Figure S3**). Since chemokines release by keratinocytes in response to environmental danger signals is critical for attracting cells of the innate and adaptive immune systems (20, 21), we assessed the role of Ncs in keratinocytes activation. The results showed that Ncs significantly reduced the secretion of CCL1/2/20 and CXCL1/10 in HaCaT cells (**Figure S4**), which were able to reduce the recruitment of T cells and neutrophils to the skin. Taken together, these results suggest that Ncs has potent anti-inflammatory properties against psoriasis *in vitro* and *in vivo*.

### Ncs targets lipid metabolism pathways

**Figure 4:**
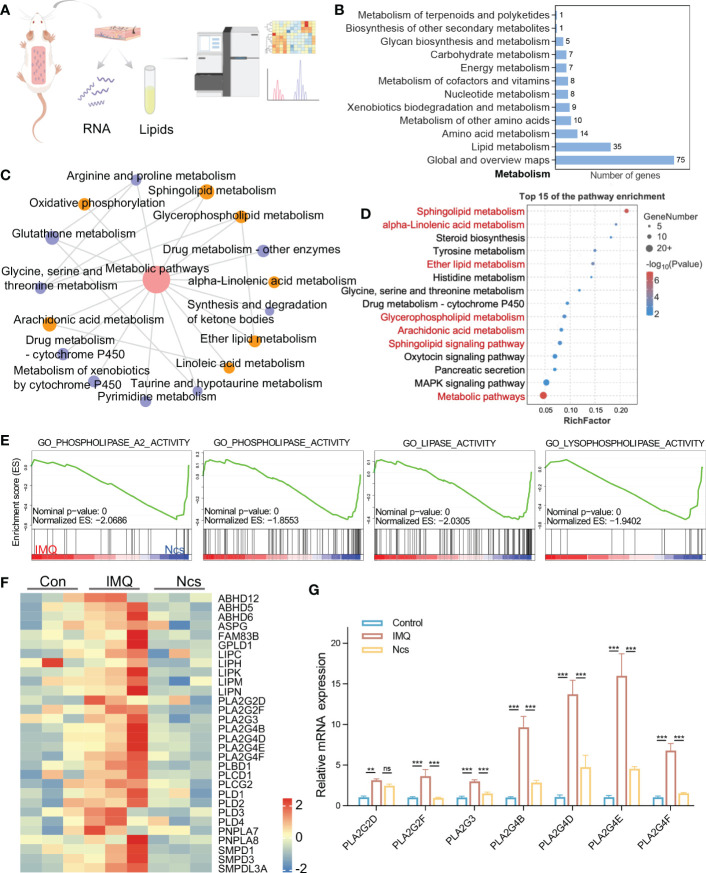
Ncs regulated genes involved in lipid metabolism especially phospholipids. **(A)** A simplified flow chart of the transcriptome and lipidomics. **(B)** Statistical graph of the number of DEGs enriched in the KEGG metabolic class. **(C)** Pathway interaction graph with p<0.05 in metabolism class, node size indicates the number of DEGs enriched in this KEEGG pathway. **(D)** The top 15 results of KEGG pathway. **(E)** GSEA enrichment plots of GO biological processes related to lipid metabolism in IMQ group and Ncs group. **(F)** Heatmaps of the DEGs from **(E)**. **(G)** PCR verification of PLA2 family genes in skin of mice (n=3) with different treatments. Data are presented as the mean ± SEM from three separate experiments. P-values were determined using t test by Prism 8; **p < 0.01; ***p < 0.001; ns, no significance.

To identify the major targets of Ncs in alleviating skin immune cell infiltration and inflammation, we performed transcriptomic analysis of skin tissues from mice in the control group, Ncs group and IMQ group using RNA-seq (**Figure 4A**). Hierarchical clustering analysis of differentially expressed genes (DEGs) showed that compared with the control group, IMQ treatment up-regulated pathways related to keratinocyte differentiation and inflammation (**Figure S5**), which was consistent with published studies (22). We further analyzed 558 DEGs (> 2-fold change) in the Ncs group versus the IMQ group and found that 230 were down-regulated due to Ncs treatment. Functional annotation analysis according to Kyoto Encyclopedia of Genes and Genomes (KEGG) pathways revealed that a large number of DEGs were clustered in metabolic class (**Figure 4B**). The interaction network diagram (**Figure 4C**) of significantly enriched pathways and the bubble diagram (**Figure 4D**) of top 15 pathways based on enrichment further emphasized the role of Ncs treatment on lipid-related metabolic processes, especially phospholipids (including glycerophospholipids and sphingolipids). In addition to being the main components of biological membranes, phospholipids and their metabolites contribute to physiology *in vivo* such as vesicle trafficking, signal transduction and molecular transport (23, 24). Gene set enrichment analysis (GSEA) was performed to identify specific gene-ontology biological processes (GOBPs) in the Ncs group and IMQ group. As expected, the IMQ group significantly up-regulated – and the Ncs group significantly down-regulated – biological process related to phospholipid metabolism (**Figure 4E**). **Figure 4F** shows the expression of key genes in the mentioned processes. Interestingly, multiple phospholipase A2 family genes were downregulated including PLA2G3, PLA2G4B/D/E/F. PLA2 specifically recognizes and catalytically hydrolyzes the sn-2 acyl bond of phospholipids, releasing arachidonic acid and lysophosphatidic acid, which participate in inflammatory reactions after downstream modification. We further validated the RNAseq results by qRT-PCR analysis as shown in **Figure 4G**. In addition, PLD family members were also observed to be reduced to varying degrees. The main substrate of PLD is phosphatidylcholine, whose hydrolyzate is involved in various physiological processes and diseases. Taken together, these results highlight the role of Ncs in lipid metabolism, especially phospholipid-related metabolic processes.

### Ncs broadly regulates phospholipid metabolism and increases anti-inflammatory lipid molecules

**Figure 5:**
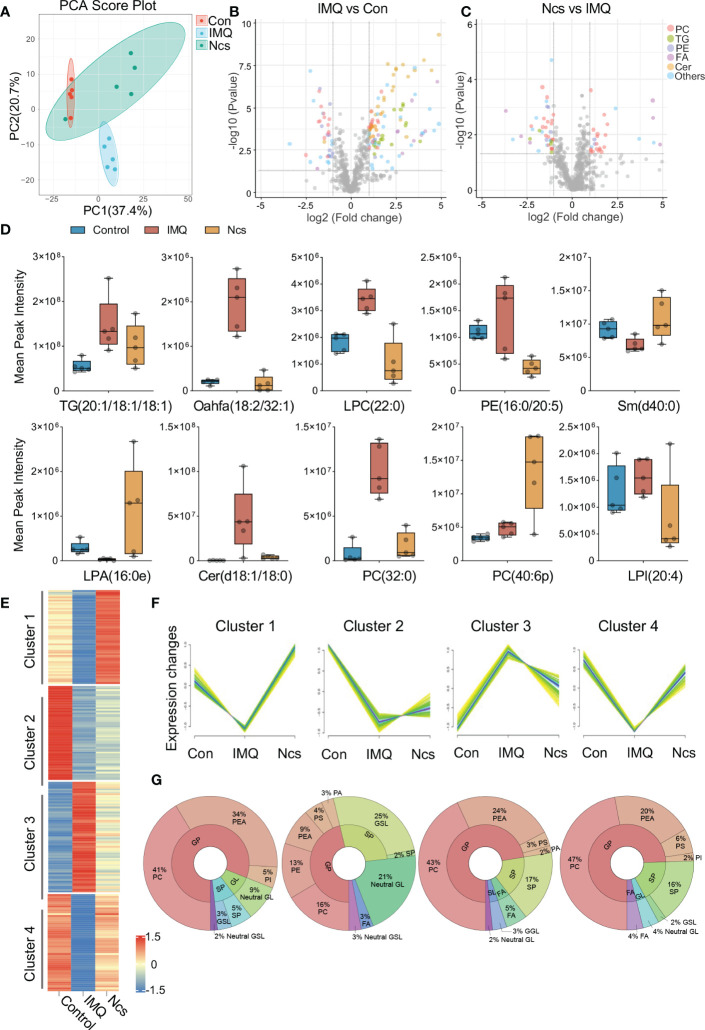
Lipidomics analysis of skin lesions in mice with different treatment. **(A)** PCA clusters samples with similar trends based on the expression profiles of lipid metabolites. The 15 samples were clearly separated into three clusters, corresponding to Con, IMQ and Ncs group (n=5). **(B, C)** Volcano plots of DELs in the comparison of IMQ vs. Con and Ncs vs. IMQ. Different colors correspond to different lipid family. **(D)** Changes of lipid metabolite levels in skin of mice treated with different treatments. **(E)** Heat map of 4 representative clusters in hierarchical cluster analysis. **(F)** trend line of DEL level in the 4 clusters from **(B)**. **(G)** Pie charts show the lipid classes and proportions of DELs in each cluster.

Considering the important role of phospholipids in regulating cellular metabolism and the suggestion of phospholipid metabolism disturbances in IMQ+ mice based on RNA-seq results, we performed lipidomic analysis of skin tissue from Ncs-treated mice. Our aim was to evaluate the effect of Ncs on therapeutic changes in the phospholipid content and other lipid species in IMQ-induced psoriasis. Principal component analysis (PCA) was first applied to analyze the overall variation of the samples. A general clustering trend across groups was observed along the PC1/PC2 score plot, with the Ncs group scattered in the area between the control and IMQ groups (**Figure 5A**). Next, we identified differentially expressed lipid metabolites (DELs) with the rule FC>=2, p<0.05. Alterations in the lipid profile of IMQ group when compared with Control (**Figure 5B**), and in Ncs group when compared with IMQ group (**Figure 5C**), are shown as volcano plots. Each lipid family is coded with a different color. We found that lipid metabolism, especially phospholipid groups, was disturbed in IMQ-treated skin tissues compared with controls. The lipid molecules in the Cer, PC, PE and PG changed to a varying degree. These findings are consistent with previous researches (25–28) that identified dysregulation of phospholipid metabolism including glycerophospholipids (PEA, PI, PC, etc.) and sphingolipids (sphingomyelin, sphingosine, etc.) in psoriasis. However, Ncs treatment restored the expression profiles of some lipid molecules to similar levels to controls. The expression of representative DELs for different lipid groups is shown in **Figure 5D**. In addition, we also found that the expression of TG in the skin tissue of IMQ group was significantly increased (**Figure 5B**), which was positively correlated with the risk of psoriasis (4), and Ncs treatment reduced this effect. Furthermore, hierarchical clustering analysis by the Mfuzz (29) software package further clustered metabolites with similar expression patterns into associated clusters based on the expression information. Among the 12 clusters obtained, 4 clusters of interest are shown in **Figure 5E**. Compared with the control group, the relative content of DELs in Cluster3 was significantly up-regulated in the IMQ group, and then reversed by Ncs (**Figure 5F**). Conversely, in Cluster1,2,4, the relative amounts of DELs were down-regulated in the IMQ group, whereas Ncs restored their levels similar to Con (**Figure 5F**). Then we found that the lipid classes and proportions of DELs in each cluster were: 16-47% phosphatidylcholine (PC), 9-34% phosphatidylethanolamine (PEA) and 5-17% sphingolipid (SP). Other components of the cluster are also shown in **Figure 5G**. Since PCs accounted for a significant proportion of DELs after Ncs treatment, we focused on differentially expressed PCs compared to IMQ. The reduced PC species in the Ncs group underwent similar changes in the Con group, whereas they were up-regulated in the IMQ group (**Figure S6**). Notably, most of the PC species up-regulated by Ncs treatment underwent structural changes whereby the alkyl group attached to the sn-1 position through a vinyl ether bond. Such modified PCs, Plasmenylcholine (PlgPC), have antioxidant properties due to their ability to scavenge free radicals (30). Taken together, these data suggested that Ncs broadly modulate skin lipid profiles and increase the content of anti-inflammatory lipid molecules.

### Ncs acts on PLA2 in keratinocytes

**Figure 6:**
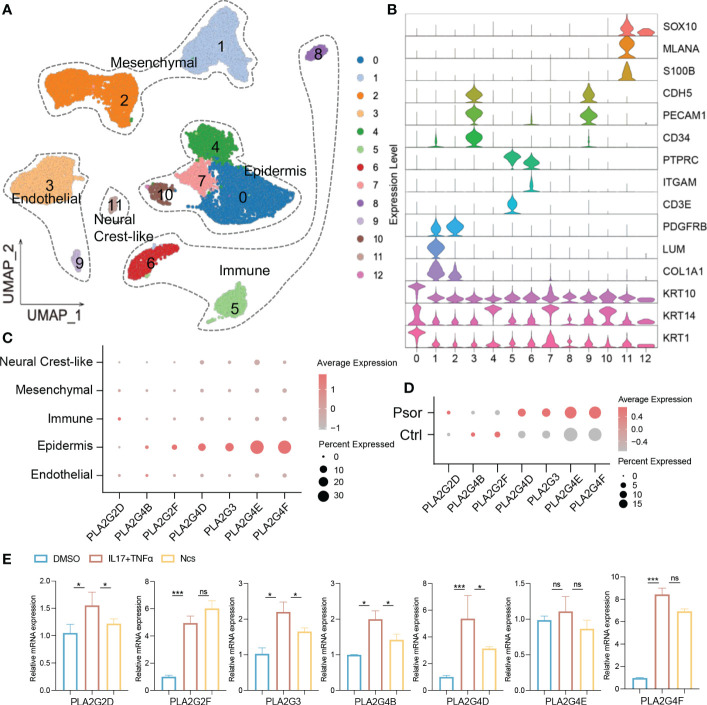
Ncs acted on the PLA2 family genes of keratinocytes. **(A)** UMAP visualization of full-thickness skin cells of 3 psoriasis patients and 3 healthy volunteers from the GSE162183 dataset. Unsupervised clustering analysis identified them as 12 broad cell types. **(B)** The violin plots displayed scaled expression of selected top significant marker genes for clusters in **(A)**. Based on the expression of marker genes, these 12 cell types were defined into 5 main clusters: endothelial (CDH5, PECAM1, CD34); epidermis (KRT10, KRT14, KRT1); immune (PTPRC, ITGAM, CD3E); mesenchymal (PDGFRB, LUM, COL1A1) and neural crest-like (SOX10, MLANA, S100B). **(C)** Dot plot of PLA2 family gene distribution for each cell type in all psoriasis and healthy donors. Dot color indicated the average expression of the gene. Size represented the percentage of cell type expressing the gene. **(D)** Dot plot of PLA2 family gene expression levels in psoriasis and healthy donors. **(E)** PCR analysis of relative expression of PLA2 family genes in not activated, IL17/TNF-activated and in activated and Ncs treated HaCaT cells. Data are presented as the mean ± SEM from three separate experiments. P-values were determined using t test by Prism 8; *p < 0.05; ***p < 0.001; ns, no significance.

Transcriptomic and lipidomic results cross-validated the effect of Ncs on phospholipid metabolism. We focused on PLA2 family genes because they account for a large proportion of differentially expressed genes. qRT-PCR confirmed that Ncs reduced mRNA expression of PLA2 in IMQ-induced mice skin (**Figure 4G**). To further localize PLA2 expression, single-cell RNA sequencing (scRNA-seq) data was used for subsequent analysis. We downloaded scRNA-seq datasets GSE162183 from the Gene Expression Omnibus (GEO) database and used the Seurat platform for clustering and visualization. A total of 18, 914 skin cells from psoriasis lesions and healthy volunteers were included for further bioinformatics analysis. Unsupervised clustering from UMAP (Uniform Manifold Approximation and Projection) depicted 12 clusters defining broad cell types present (**Figure 6A**). Combined with differentially expressed genes and established canonical markers (**Figure 6B**), these 12 clusters were identified into five major cell groups, which were Mesenchymal, Immune, Neural Crest-like, Epidermis and Endothelial cells (**Figure 6A**). The normalized expression levels of PLA2 in each cell population indicated that it was predominantly expressed in keratinocytes and was rarely detected in other constituent cell types of skin (**Figure 6C**). In addition, we further confirmed that PLA2 was commonly elevated in psoriasis, although the expression levels of PLA2G4B and PLA2G2F were incompatible with some previous studies, possibly due to sample size and individual variability (**Figure 6D**). We then stimulated HaCaT cells *in vitro* with the pro-inflammatory cytokine combination of TNFα and IL17A and examined the effect of Ncs on PLA2. We observed that the application of TNFα and IL17A induced high expression of PLA2, whereas the use of Ncs reduced PLA2 family genes to varying degrees (**Figure 6E**).

## Discussion

Psoriasis is a skin disease that involves multiple organs. The association between psoriasis and metabolic syndrome including obesity, diabetes, dyslipidemia, and hypertension suggests a role for lipid metabolism disorders in the pathogenesis of psoriasis (3, 4). Thus, therapeutic modalities that improve lipid metabolism could both reduce the inflammatory burden of psoriasis and reduce the risk of metabolic disease. In this study, we provide the first evidence that Ncs can target lipid metabolism, especially phospholipid metabolism, to ameliorate IMQ-induced psoriasis-like dermatitis in mice.

Our study found that the application of Ncs broadly altered lipid metabolism genes in IMQ-induced psoriasis model. In particular, both KEGG and GSEA analysis revealed significant enrichment of differential genes in pathways related to phospholipid metabolism. In addition to being a major structural component of cell membranes, phospholipids regulate a variety of biological processes including cell proliferation, apoptosis, immunity, angiogenesis, and inflammation (31, 32). Phospholipase A2 (PLA2) catalyzes the hydrolysis of the sn-2 acyl bond of glycerophospholipids and releases pro-inflammatory lipid mediators and free fatty acids (33, 34). Application of Ncs down-regulated multiple members of the PLA2 family including PLA2G2F, PLA2G4B/D/E/F. Of these, PLA2G2F is the best studied in skin homeostasis, where it has been proven to be important in hyperproliferative epithelial diseases such as psoriasis and skin cancer (7, 35). Inhibition of PLA2G4D/E could regulate keratinocyte aberrant differentiation and immune responses (8). PLA2G4D-containing exosomes released by mast cells can activate T cells and induce IL-22 and IL-17A to promote psoriatic inflammation (36). Inhibitors targeting cytosolic PLA2 have shown a favorable efficacy and safety in phase I/IIa clinical trials as therapeutic agents for plaque psoriasis (37). Our results suggested that Ncs may exert therapeutic effects through a similar mechanism in psoriasis. In addition, Ncs exhibited inhibitory effects on members of the phospholipase D(PLD) family. PLD hydrolyzes phosphatidylcholine (PC) to produce signaling lipids (PtdOH or PA) and free choline (38). Aberrant PLD/PtdOH signaling activates phosphodiesterase (PDE) 4 family members in a Camp-dependent manner (38), which further activates cascade of several signaling steps (e.g., PKA/CREB/NFκb), leading to increased proinflammatory cytokines TNF-α, IL-23, IFN-β and γ and promote psoriasis progression (39, 40).

The lipidomic analysis and RNA-seq results interactively verified the regulatory effect of Ncs on lipid metabolism, especially phospholipid. Previous studies have shown significant changes in plasma lysoglycerophospholipids (such as LPA and LPC) and glycerophospholipid metabolism (including PA, PC, and PI) in patients with psoriasis. In addition to restoring some lipid molecules to similar levels to controls, our results showed that Ncs strongly increased anti-inflammatory plasmalogens. Dysregulation of plasmalogen metabolism is associated with the development of obesity-related metabolic disease and inflammatory bowel disease (41–43). Rescue of its levels prevents hepatic steatosis and NASH (non-alcoholic steatohepatitis) by increasing fatty acid oxidation through PPARα signaling (44). Furthermore, plasmalogen is degraded by PLD or PLA2 to generate free fatty acids that participate in inflammatory responses (45, 46). Our results found that Ncs treatment extensively reduced the expression of phospholipase family members, which may account for the increased content of anti-inflammatory plasmalogens.

Overlapping signaling molecules in lipid metabolism disturbances and inflammatory responses link their pathophysiology. Lysophospholipids and free fatty acids (especially arachidonic acid) are the main components of the catalytic products of PLA2. Among them, lysophosphatidylcholine (lysoPC) can stimulate leukocyte activation, T lymphocyte chemotraction and inflammatory cell accumulation (47, 48). Prostaglandins (PG) and leukotrienes (LT) derived from PLA2-mediated “arachidonic acid cascade” can activate NF-κB through the TNF-signal transduction pathway, thereby effectively promoting keratinocyte division and activation (49–51). These findings may associate the suppressive effect of Ncs on keratinocyte proliferation and the attenuated Th17 response in our results with PLA2.

Collectively, our results demonstrate that Ncs alleviates IMQ-induced psoriatic symptoms through a mechanism involving PLA2 inhibition and improved phospholipid metabolism. Despite some limitations, such as the urgent need for more experimental evidence on how PLA2 regulates immune responses in keratinocytes and Th17 cells, our results provide new insights into the therapeutic effect of Ncs in psoriasis.

## Data availability statement

The data presented in the study are deposited in the Genome Sequence Archive repository in National Genomics Data Center, China National Center for Bioinformation/Beijing Institute of Genomics, Chinese Academy of Sciences, accession number PRJCA013712.

## Ethics statement

All animal experiments were conducted according to the guidance of China Animal Welfare Legislation, and approved by the ethical institutional review board of Union hospital, Huazhong University of Science and Technology.

## Author contributions

YK and JJ designed the study and drafted the original manuscript. YH, LL and XL helped modify the figures. ZJ and FW helped with flow cytometry. XXL and JY analyzed the data. YZ, QT and HC offered constructive comments on the manuscript. All authors contributed to the article and approved the submitted version.
