# Supplementary material for: Narciclasine inhibits phospholipase A2 and regulates phospholipid metabolism to ameliorate psoriasis-like dermatitis
Source: Front Immunol. 2023 Jan 4;13:1094375. doi: 10.3389/fimmu.2022.1094375 (PMC9869703; doi:10.3389/fimmu.2022.1094375)
Supplement: Supplementary file 2 [file DataSheet_1.docx]

**Figure S1. Ncs decreased inflammatory molecules in IMQ-induced psoriatic mice.** (A) The relative mRNA levels of IL1β, IL6, IL10, IL17A, IL23, IFNγ and TNFα were determined by PCR in skin of mice with different treatment. (B) The expression levels of TNFα, IFNγ, IL6, IL10 and CCL2 in mice serum were detected by flow cytometry using CBA kit. Data are presented as the mean ± SEM. P-values were determined using t test by Prism 8; **p* < 0.05; ***p* < 0.01; ****p*< 0.001.

**Figure S2. Ncs reduced the proportion of CD3+CD4+ T cells in the spleen of IMQ-induced psoriasis-like mice.** The experimental methods can refer to Fig. 2. Data are presented as the mean ± SEM. P-values were determined using t test by Prism 8; **p* < 0.05; ***p* < 0.01.

**Figure S3. Effects of Ncs on apoptosis of HaCaT cells.** HaCaT cells were treated with 0.1μM Ncs or DMSO for 24h with or without 10ng/ml LPS, then stained with Annexin V-FITC/propidium iodide, and apoptosis was analyzed by flow cytometry.

**Figure S4. Ncs inhibited LPS-induced HaCat cells activation.** LPS-primed HaCaT cells were treated with or without 25nM Ncs for 24h. The relative mRNA levels of CCL1, CCL2, CCL20, CXCL1, CXCL10, S100A8 and S100A9 were determined by PCR in skin of mice with different treatment. Data represent the mean ± SEM from three independent experiments. P-values were determined using t test by Prism 8; **p* < 0.05; ***p* < 0.01; ****p* < 0.001.


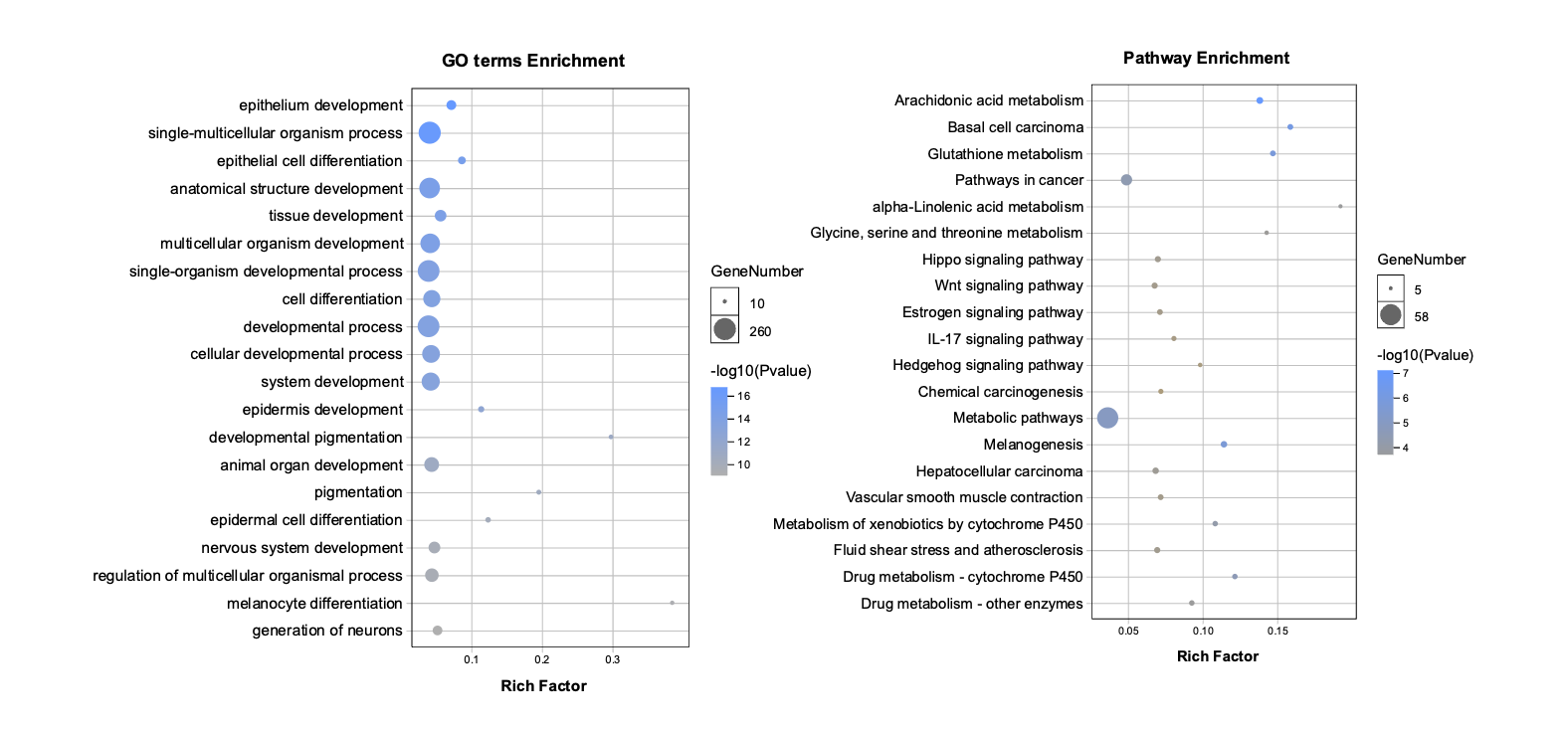
**Figure S5. GO and KEGG enrichment analysis of DEGs between IMQ and Con group.**

**Figure S6. Heatmap of differentially expressed PCs induced by Ncs, compared to IMQ treatment.** The letter P marked in red represents Plasmenylcholine (PlgPC), a structural change in the lipid molecule where the alkyl group is linked to the sn-1 position by a vinyl ether bond.
